# Posterior Circulation Occlusions May Be Associated with Distal Emboli During Thrombectomy: Factors for Distal Embolization and a Review of the Literature.

**DOI:** 10.1007/s00062-018-0679-z

**Published:** 2018-03-22

**Authors:** Leonard L. L. Yeo, Ake Holmberg, Anastasios Mpotsaris, Michael Söderman, Staffan Holmin, Asa Kuntze Söderqvist, Marcus Ohlsson, Pervinder Bhogal, Vamsi Gontu, Tommy Andersson, Patrick A. Brouwer

**Affiliations:** 1 Department of Clinical Neuroscience, Karolinska Institutet and Department of Neuroradiology, Karolinska University Hospital, Solnavägen 1, 171 77 Solna, Stockholm, Sweden; 2 Division of Neurology, Department of Medicine, National University Health System, Singapore, Singapore; 3 Department of Neuroradiology, University Hospital of Aachen, Aachen, Germany; 4 Neuroradiology Clinic, Klinikum Stuttgart, Kriegsbergstraße 60, 70174 Stuttgart, Germany; 5 Department of Medical Imaging, AZ Groeninge, 8500 Kortrijk, Belgium

**Keywords:** stroke, Emboli, Complication, Thrombectomy

## Abstract

**Background:**

Distal embolization or movement of the thrombus to previously uninvolved vasculature are feared complications during stroke thrombectomy. We looked at associated factors in a consecutive series of patients who underwent thrombectomy with the same endovascular device.

**Methods:**

We included all patients with acute ischemic stroke in the anterior or posterior circulation, who underwent thrombectomy with the same thrombectomy device for acute stroke from 2013 to 2016. Distal embolization was defined as any movement of the thrombus into a previously uninvolved portion of the cerebral vasculature or the presence of thrombotic material further downstream in the affected vessel, which occurred after the initial angiogram. We studied patient-related as well as technical factors to determine their association with distal emboli.

**Results:**

In this study 167 consecutive acute stroke patients treated with the emboTrap® device (Cerenovus, Irvine, CA, USA) were included with a median National Institutes of Health Stroke Scale (NIHSS) of 15 (range 2–30) and mean age of 67 years (SD 13.1 years). Of the patients in our cohort 20 (11.9%) experienced distal emboli, with 2.3% into a new territory and 9.6% into a territory distal to the primary occlusion. On univariate analysis, age, intravenous tissue plasminogen activator (tPA), posterior circulation occlusions, and general anesthesia were associated with distal emboli. On multivariate analysis, only posterior circulation occlusions (odds ratio OR 4.506 95% confidence interval CI 1.483–13.692, *p* = 0.008) were significantly associated with distal emboli. Distal embolization was not significantly associated with worse functional outcomes at 3 months, increased mortality or increased bleeding risk.

**Conclusion:**

Posterior circulation occlusions were significantly associated with distal emboli during thrombectomy, possibly due to the lack of flow arrest during such procedures. New techniques and devices should be developed to protect against embolic complications during posterior circulation stroke thrombectomy.

## Introduction

Clot retrieval systems have been proven to be an effective treatment in acute ischemic stroke (AIS) with high rates of recanalization; however, the rates of good clinical outcomes have not always mirrored this success [1–4]. There are several possible causes of divergence and one suggested cause is that clot retrievers can cause fragmentation of the thrombus with the release of distal emboli [5, 6]. Procedure-related embolic complications have been noted with various thrombectomy devices [7–10]. These clots can block collateral flow to potentially salvageable tissue or even cause ischemia in a previously unaffected territory [11–14]. The presence of these fragmented clots has been shown to be associated with worse clinical outcomes [12, 15]. Nonetheless, procedural release of embolic particulate matter during mechanical thrombectomy is a modifiable risk factor that may be minimized with correct tools and techniques.

We studied a core laboratory adjudicated stroke database to determine if we could identify factors associated with embolic complications distal to the initial occlusion as well as to previously unaffected territories during mechanical thrombectomy.

## Methods

All consecutive AIS patients treated with the emboTrap® thrombectomy device between October 2013 and November 2016 were assessed. Inclusion criteria were large vessel occlusion and computed tomography (CT) perfusion assessed viable tissue, regardless of time of onset, and treatment with the emboTrap® revascularization device. Patients arriving within the 4.5 h time window received intravenous thrombolytic treatment where indicated. Signs of manifest infarction on CT-perfusion was not an exclusion criterion if there was still viable tissue to be saved. We did not use the absolute volume of infarction or the ischemic/hypoxic ratio as exclusion criteria as this, in our view, may exclude patients who might benefit from thrombectomy [16]. For the anterior circulation, an 8 or 9 French balloon guide catheter (BGC, Merci™ or FlowGate™, Stryker, Kalamazoo, MI, USA) was typically placed in the cervical internal carotid artery via an 80 cm Arrow sheath (Teleflex®, Wayne, Penn, USA). A 0.021-inch microcatheter was navigated through the occlusion and the emboTrap® deployed at the site of the clot. In the anterior circulation, a 6 French intermediate catheter (IM) was sometimes used, based on operator preference and positioned just proximal to the occlusion whereupon the emboTrap® was fully or partially retrieved into the IM under aspiration in both the IM and the BGC. In the posterior circulation, a 6 French Envoy guide catheter or intermediate catheter was placed, via a 65 cm Arrow sheath, into the dominant vertebral artery, after which the emboTrap® was placed through a 0.021-inch microcatheter. Aspiration on the guiding catheter was done during the entire retrieval of the emboTrap® device, most intensively while passing into the guide or intermediate catheter.

**Fig. 1:**
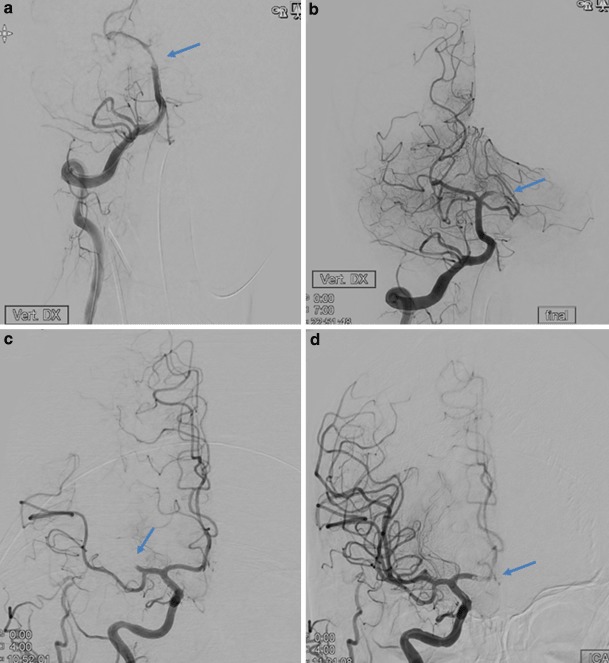
Computed tomography angiography. **a** and **b** Distal embolization in the same vascular territory: a angiographic run showing basilar artery occlusion (arrow showing location of occlusion), **b** after thrombectomy there is a left posterior cerebral artery thrombus (arrow showing new occlusion). **c** and **d** Distal emboli in a previously unaffected territory. **c** Right middle cerebral artery occlusion (arrow showing MCA occlusion). **d** After thrombectomy there is now an anterior cerebral artery thrombus (arrow showing new occlusion in the ACA)

We hypothesized that certain variables, such as not ensuring flow arrest or the absence of intravenous tPA would be associated with embolic complications. We studied the following factors to determine if there was such an association: age, gender, the use of intravenous tPA, recent stroke, location of the occlusion, the use of general anesthesia, the National Institutes of Health Stroke Scale (NIHSS) score, the use of an intermediate catheter, the modified Thrombolysis in Cerebral Infarction (mTICI) score at the end of the procedure, the procedural duration and the number of thrombectomy attempts. The modified Rankin Scale (mRS) score at 3 months, the time intervals from onset of symptoms to imaging, groin puncture, and reperfusion were collected. Filling defects present on final angiographic runs after the retrieval, but absent on preceding computed tomography angiography (CTA) or the initial angiographic runs were considered to be new distal emboli, whether distal to the occlusion or in new territories (Fig. 1.) We used this definition to ensure all such distal emboli were recorded, as compared to other studies where only emboli in new territories were considered as a complication [1, 2]. The pre-treatment CTA was reviewed to ensure that no distal emboli were present. All post-procedural images were core laboratory assessed by neuroradiologists from a different country uninvolved in the patient treatment and blinded to the clinical factors. They looked for secondary emboli distal to the occlusion, emboli in previously unaffected territories and mTICI score [17]. Although successful recanalization was defined as a score greater than or equal to 2b, when to stop the procedure was left to the operator’s discretion. Good functional outcome was defined as mRS ≤2 at 3 months. All mRS results were scored by independent neurologists in a national database. Symptomatic intracranial hemorrhage (sICH) was defined by the core lab as any hyperdensity with a worsening of NIHSS by 4 points or more [18].

### Statistical Methods

We presented the numerical variables as mean and standard deviation or median and range. Categorical variables are presented as percentages. Numerical predictors were tested by using 2‑sample *t-*test or Mann-Whitney *U-*test where applicable. Categorical variables were evaluated using χ^2^-test or Fisher’s exact test where applicable. Variables that were found to have a significant association (*p* < 0.05) were entered into the multivariable model to perform logistic regression for determining the independent predictors of embolic complications. Associations were presented as odds ratios (OR) with corresponding 95% confidence intervals (CI). Statistical analyses were performed using the Statistical Package for Social Sciences (SPSS) version 21.

Institutional ethics review board approval for this study was obtained from the regional ethical committee in Stockholm (Regionala Etikprövningsnämnden i Stockholm, diarienr:2016/1041-31/4). This research received no specific grant from any funding agency in the public, commercial or not-for-profit sectors.

## Illustrative Case Descriptions

**Fig. 2:**
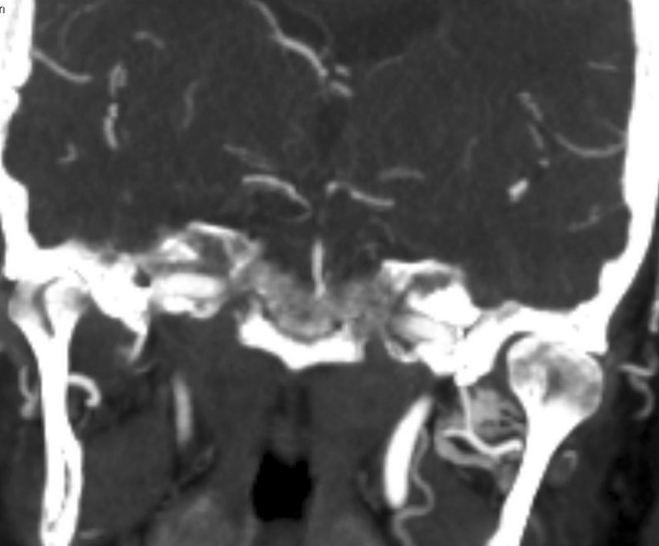
A computed tomography angiogram pre-treatment in the same patient as Fig. 1a and b, showing that the left posterior cerebral artery was patent before the procedure

The first middle-aged patient presented with giddiness then subsequently loss of consciousness. A CT-angiogram was performed showing the basilar occlusion and the patent posterior cerebral artery (Fig. 2). Using a right femoral access, an Envoy guide catheter was placed via a 65 cm Arrow sheath into the vertebral artery and a Prowler® Select® Plus (Codman & Shurtleff, Inc., Raynham, MA, USA) microcatheter was advanced past the clot. An emboTrap® 5 × 33 was deployed and the clot was removed after one pass however the subsequent angiogram showed distal embolization of part of the clot into the same vascular territory (Fig. 1a and b). Post-procedure the patient recovered well but had neurological sequela of a visual field defect. The second middle aged patient had acute left sided weakness and neglect from the right middle cerebral artery occlusion (Fig. 1c), the patient was treated with a long sheath through which a balloon guide catheter and a prowler select plus microcatheter delivered an emboTrap® 5 × 33 device. After the first pass, part of the thrombus had migrated into the right anterior cerebral artery (Fig. 1d). This was then removed with another pass of the emboTrap® 5 × 33 and the patient recovered well.

## Results

A total of 167 consecutive patients were included with 56.8% being male and with a mean age of 67 (SD 13.1) years. The median pre-treatment NIHSS score was 15 (range 2–30). The location of the occlusion was in the anterior circulation in 88.0% and in the posterior circulation in 13.8%, including 3 patients having occlusions in both. Of the patients 78 (46.7%) received intravenous tPA before the procedure and mTICI 2b/3 was achieved in 141 (84.4%) patients. (Table 1). Of the patients 20 (12.0%) had distal secondary emboli, of which 2.4% were in a new territory and 9.6% were in the same vascular territory, distal to the initial occlusion. Out of 147 patients with anterior circulation stroke, 13 (8.9%) had distal emboli and out of the 23 with posterior circulation stroke, 7 had distal emboli (30.4%). Good functional outcome at 3 months was seen in 85 patients (50.9%), of which 79/146 (54.1%) were in the anterior circulation and 7/23 (30.4%) in the posterior circulation including 1 patient having an occlusion in both the anterior and posterior circulation. Out of 167 patients 5 suffered from sICH (3.0%) while 21 patients (12.6%) were deceased at 3 months follow-up and 2/23 (8.7%) patients with posterior large vessel occlusion had SICH and 8/23 (34.8%) died. Of the patients 19 were lost to follow-up.

**Table 1:** Baseline characteristics and results of the study population

Characteristic	Number (%)
Mean age (SD) (years)	67 (13.1)
Male sex	95 (56.8%)
Median pre-treatment NIHSS score (range)	15 (2–30)
General anesthesia	23 (13.8%)
Conscious sedation	144 (86.2%)
Intravenous tPA	78 (46.7%)
Recent stroke	6 (3.6%)
Intermediate catheter use	41 (27.9%)
Anterior circulation occlusion	147 (88.0%)
Posterior circulation occlusion	23 (13.8%)
Anterior cerebral artery occlusion	4 (2.7%)
Internal carotid artery occlusion	31 (21.1%)
Middle cerebral artery occlusion	94 (63.9%)
Terminal internal cerebral artery occlusion	18 (12.2%)
Basilar artery occlusion	17 (10.2%)
Vertebral artery occlusion	6 (3.6%)
Puncture to reperfusion time (SD) (mins)	66.38 (46.57)
Distal emboli	20 (12.0%)
mTICI 2b/3	141 (84.4%)
Mortality	21 (12.6%)
Modified Rankin Scale ≤ 2 at 3 months	85 (50.9%)
Symptomatic intracranial hemorrhage	5 (3.0%)

On univariate analysis, the absence of intravenous tPA, older age, general anesthesia and posterior circulation occlusions were significantly associated with distal emboli (Table 2). On multivariate analysis, only posterior circulation occlusions were significantly associated with distal emboli (OR 4.506, 95%CI 1.483–13.692, *p* = 0.008). The use of intravenous tPA (OR 2.447, 95%CI 0.808–7.407, *p* = 0.113), older age (OR 1.035 per year, 95%CI 0.989–1.052, *p* = 0.134) and general anesthesia (OR 2.534, 95%CI 0.718–8.941, *p* = 0.148) failed to reach significance.

**Table 2:** Results of univariate and multivariate analysis: predictors of distal emboli. Effect of distal emboli on outcomes (mRS 0–2, sICH, mortality) at the bottom of the table

	Univariate analysis			Multivariate analysis	
	Distal emboli (*n* = 20)	No distal emboli (*n* = 147)	*P*-value	Adjusted odds ratio (95% C.I.)	*P*-value
Age, mean (SD), year	71.8 (9.36)	66.3 (13.41)	*0.026*	1.04 (0.99–1.05)	0.134
Male	12 (60.0%)	83 (56.5%)	0.480	–	–
Intravenous tPA	6 (30.0%)	73 (49.7%)	**0.038**	0.411 (0.13–1.26)	0.119
Recent stroke	1 (5.0%)	6 (4.1%)	0.543	–	–
Posterior circulation occlusion	7 (35.0%)	16 (10.9%)	*0.009*	*4.51 (1.48–13.69)*	*0.008*
General Anesthesia	7 (35.0%)	16 (10.9%)	*0.009*	2.53 (0.72–8.94)	0.148
NIHSS at onset, median (range)	18 (3–30)	14 (2–26)	0.276	–	–
Intermediate catheter	8 (40.0%)	33 (22.4%)	0.080	–	–
TICI 2b/3	16 (80%)	125 (85.0%)	0.339	–	–
Number of attempts, median (range)	3 (1–6)	2 (1–10)	0.073	–	–
Puncture-to-reperfusion time, mean, (SD)/median, min	79 (45.2)/53.3	76 (47.2)/54.2	0.670	–	–
*Outcomes*					
mRS 0–2	8 (40%)	77 (52.4%)	0.346	–	–
sICH	3 (15%)	2 (1.3%)	0.013	–	–
Mortality	5 (25%)	16 (10.8%)	0.084	–	–

*tPA* tissue plasminogen activator, *NIHSS* national institute of health stoke scale, *TICI* thrombolysis in cerebral infarction scale, *mRS* modified Rankin scale, *sICH* symptomatic intracranial haemorrhage

Finally, the presence of distal emboli was not significantly associated with poor functional outcomes at 3 months (OR 0.623 95% CI 0.241–1.612, *p* = 0.229); however, there was a trend towards increased mortality (OR 2.73 95% CI 0.875–8.512, *p* = 0.084) and distal emboli was significantly associated with sICH (OR 12.794 95% CI 1.995–82.05, *p* = 0.013). Nonetheless, due to the limited incidence of sICH, the 95% confidence intervals are wide.

## Discussion

Our study showed an association between posterior circulation occlusions and distal embolization. These distal emboli were in turn associated with sICH and with a trend towards increased mortality. Secondary distal embolization to the same or previously unaffected territories is a feared complication during thrombectomy, which may reduce blood flow to the affected territory as well as inhibit collateral flow. This may result in penumbral tissue loss and an increased ischemic area [19]. Previous studies have confirmed that the presence of multiple distal emboli is predictive of worse functional outcomes [20]. In vitro experiments have demonstrated that large amounts of clot fragments can be released during a thrombectomy procedure, but the majority of these clot fragments are small, approximately 10 μm in size. The resulting small occlusions may not be appreciated on standard digital subtracted angiography and the actual incidence of distal emboli could be higher than reported [21, 22].

Hypothetically there are several factors which can generate emboli during thrombectomy: 1) the initial breaking or even “grating” of the clot by the mesh of a thrombectomy device as it is deployed, 2) the opposing shear forces of the blood flow breaking pieces off of the clot and 3) the friction of vessel wall and ostia of the side branches which may fragment the thrombus as it is withdrawn [6], 4) when a thrombectomy device is pulled into a larger vessel from a smaller vessel it expands due to the intrinsic tension and this can result in a temporary loss of apposition between most devices and the thrombus and finally, 5) shearing off of the sides of the clot by the edges of the guide catheter as the clot is pulled into it. The device used in our study (emboTrap®) is specifically designed to minimize these mechanisms and be more effective than the previous generations of devices, especially the standard stent retrievers without a distal protection coil. Nonetheless this efficacy remains to be proven as there is not yet a trial comparing the emboTrap® to other thrombectomy devices.

To illustrate the magnitude of the problem, the MR CLEAN study, in which a variety of different thrombectomy techniques were used, had thrombus embolization to new territories in 8.6% of the patients which resulted in 5.6% developing new neurological deficits. [1]. Older thrombectomy devices which fragment the clot more frequently like the MERCI device or the initial iteration of the Penumbra system (Penumbra, Inc., Alameda, CA, USA) resulted in poorer outcomes than even intra-arterial tPA thrombolysis, presumably due to a high embolic load, and have fallen out of favor [22–25]. For studies predominantly using stent retrievers, 5–22% had distal emboli with 0–7% of these into new vascular territories [1, 2, 10, 11, 26–32]. The direct aspiration technique has an embolic rate as high as 16% under clinical conditions ([33, 34], Table 3). The recent ASTER trial, however, showed no statistically significant difference between the two techniques for emboli to new territories bearing in mind that they only included anterior circulation occlusions [35]. Our study showed a lower incidence of distal emboli to new territories of 2.3%. Our study did not show an association between distal emboli and poorer outcomes, but it did show an association with SiCH.

**Table 3:** Summary of thrombectomy trials and distal emboli

Study or author’s name	Thrombectomy device	*N*	Posterior circ occ (*N*)?	Anterior circ BCG use?	Emboli distal to occlusion	Emboli in new territory	Date of study	Core-lab assessed distal emboli?
Current study	emboTrap®	167	Yes (23)	Yes	9.6% (6.6% anterior, 3% posterior circ)	2.4% (1.2% anterior 1.2% posterior circ)	2017	Yes
MR CLEAN [1]	MR CLEAN	233	No	Unknown	x	8.6%	2015	Yes
EXTEND-IA [2]	Solitaire	35	No	Unknown	x	6%	2015	No
ADAPT-FAST [7]	Penumbra	100	Yes (5)	No	10%	0%	2014	No
Dorn et al. [10]	Phenox (79.8%) and Solitaire	104	Yes (24)	Intermediate Cath only	x	3.7%	2012	No
Akins et al. [11]	Solitaire (61.2%) and merci	144	Yes (4)	No	4.9%	0.7%	2014	Yes
Kurre [12]	Various devices	105	No	Intermediate Cath only	x	11%	2013	No
Gascou et al. [28]	Solitaire	144	Yes (35)	Yes	Embolic complication 12.5%		2014	No
Nguyen et al. [29]	Solitaire	338	Yes (35)	Yes	18%	5%	2014	No
Mazur et al. [30]	Solitaire	39	Yes (8)	Yes	15%	8%	2016	No
RECOST [31]	Solitare	50	Yes (16)	Yes	x	8%	2011	No
Nogueira et al.[32]	Merci/Trevo	178	Yes (12)	Yes	x	4%/7%	2012	Yes
ASTER [35]	Aspiration/stent retriever	181/176	No	Yes	X	5.3%/8.5%	2017	Yes
Menon [40]	Penumbra	27	Yes (5)	No	48%	x	2011	No
THRACE [42]	Stent retriever 83%, aspiration 16%	204	Yes (2)	Unknown	x	6%	2016	Yes

*N* numbers, *circ* circulation, *occ* occlusion, *Cath* catheter, *BGC* balloon guide catheter

Stent retrievers can also be used in combination with balloon guide catheters (BGC), which can be inflated to arrest anterograde blood flow and prevent emboli from travelling distally. Studies have shown that the use of BGC reduced distal emboli, prevented emboli in new unaffected areas and was associated with good functional outcomes [5, 33, 36, 37]. Other techniques have also been shown to reduce the incidence of distal emboli. For example, more distal deployment of stent retrievers can reduce distal emboli especially if the stent retriever is designed to function in that way [38]. The PROTECT technique which uses both a BCG and distal aspiration at the clot in addition to the stent retriever can also reduce thrombus fragmentation [39]. We used a BGC, with or without a concomitant intermediate catheter as per the operator’s preference, in all patients with anterior circulation occlusions and we are therefore unable to determine if BGCs were associated with less emboli than non-BGC patients from the data in this study. Intermediate catheter use was not associated with distal emboli.

Flow reversal could not be achieved in the posterior circulation, which could explain the higher association with distal emboli in the posterior circulation occlusions compared to the anterior circulation occlusions found in this study. This is in accordance with the literature where studies which have a substantial population of posterior circulation strokes seem to have higher rates of embolic complications, [27, 28, 30, 31, 40] with the exception of the study by Dorn et al. ([10]; Table 3).

It is also suggested in the literature that patients who underwent thrombectomy with conscious sedation have more distal emboli than those under general anesthesia, possibly because of patient movement with associated technical difficulties [41]. In our study general anesthesia conversely showed a trend towards more distal emboli but this did not reach statistical significance when controlled for other variables. This could be explained by the fact that we performed the vast majority of our cases with conscious sedation. Patients with posterior circulation stroke were more likely to require general anesthesia and a direct comparison between these two major anesthetic methods becomes invalid because of a clear inclusion bias.

Finally, the number of attempts during mechanical thrombectomy has been reported to be associated with a larger number of distal emboli generated during the procedure [36]. We do not know if this is based on the type of clot or the thrombectomy technique itself. In our study, there was no association between the number of attempts and distal emboli. Intravenous thrombolysis with tPA may theoretically reduce the number of distal emboli during mechanical thrombectomy by dissolving them or conversely make the clot more vulnerable to fragmentation during thrombectomy; however, the in vivo effect of tPA on emboli has, to the best of our knowledge, not yet been studied. In our study intravenous tPA was associated with distal emboli on univariate analysis but this did not reach statistical significance in the multivariate analysis. Older age can be result in more tortuous vasculature with an increase in embolic complications when the thrombectomy device is withdrawn, in our study there was an association between distal emboli and older age but this too did not reach significance in the multivariate analysis.

Some limitations of our study need to be mentioned. This was a large single center study with no strict inclusion criteria for treatment or uniform treatment protocol enforced. We did not analyze how the various techniques, such as distal aspiration with the intermediate catheter would affect the embolization rate as these data were not available in our database. The operators had different levels of experience with the device used. Of the patients 19 did not have 3‑month mRS scores available. The strengths of the study include a realistic patient population and although the study was not a randomized clinical trial, the core-lab evaluation helped to ensure a high level of quality in the data. Finally, we used a stricter definition of distal emboli to ensure our core-lab captured all instances of distal emboli during the procedure although we do acknowledge that there was no intra-reader or inter-reader correlation of the core lab assessment of distal emboli. Moreover, we acknowledge that small occlusions cannot be detected on CTA if there are occlusions, as this modality requires blood flow pass the occlusion to allow sufficient intraluminal contrast media to show the thrombus fragments. The rate of distal embolization due to mechanical thrombectomy may therefore be lower than described and is a major limitation. An ideal scenario would have been to use an additional modality such as susceptibility weighted imaging (SWI) sequences on MRI to confirm these emboli [20].

## Conclusion

Posterior circulation occlusions are significantly associated with distal emboli in the same and previously unaffected territories during thrombectomy, likely due to the lack of flow arrest during such procedures. New techniques and devices should be developed to protect against embolic complications during posterior circulation stroke thrombectomy.
